# Pudendal Nerve Irritation as Unique Symptom of Pelvic Congestion Syndrome

**DOI:** 10.1155/crnm/7952359

**Published:** 2025-06-03

**Authors:** Christos Dimopoulos, Sotirios Bisdas, Theodosios Bisdas

**Affiliations:** ^1^Department of Vascular Surgery, Athens Medical Center, Athens, Greece; ^2^Department of Neuroradiology, Athens Medical Center, Athens, Greece

## Abstract

Pelvic congestion syndrome (PCS) is an underdiagnosed but not rare cause of chronic pelvic pain, affecting approximately 10%–30% of women of reproductive age. It is characterized by venous insufficiency and dilation of the ovarian and pelvic veins, often presenting with symptoms that worsen during menstruation or prolonged standing, and improve in the supine position. Dyspareunia and a sensation of pelvic heaviness are also frequently reported. Neurological manifestations—such as pudendal or femoral nerve irritation—are rare but may offer key diagnostic clues. We present a case of a 30-year-old woman with right-sided pelvic pain radiating to the groin and proximal thigh, consistent with neural irritation. Magnetic neurography revealed dilated pelvic veins in close proximity to the right psoas muscle and the L5 nerve root, suggesting perineural venous engorgement. Selective venography confirmed bilateral ovarian vein insufficiency, and the patient underwent successful embolization with Ruby coils and adjunct sclerotherapy. Postoperative recovery was uneventful, with complete resolution of symptoms. Follow-up at 1 year showed no recurrence, and the patient later achieved a successful pregnancy. This case highlights the potential for pelvic venous congestion to mimic or cause neural symptoms and emphasizes the diagnostic value of magnetic neurography in complex pain presentations. Endovascular treatment proved safe and effective, even in cases with neurological involvement.

## 1. Introduction

Pelvic congestion syndrome (PCS) is an underdiagnosed vascular condition and a well-recognized cause of chronic pelvic pain in premenopausal women. It is estimated to affect 10%–30% of women of reproductive age, with symptoms often worsening during menstruation or prolonged standing, and typically improving in the supine position. Dyspareunia and heaviness in the pelvis are also frequently observed [1, 2]. The underlying pathophysiology involves venous insufficiency and dilation of the ovarian and pelvic veins, leading to venous stasis and local tissue congestion [3]. The condition may be overlooked due to the nonspecific nature of symptoms and lack of awareness among clinicians. Gynecologists usually see patients who present with chronic pelvic pain to rule out endometriosis or other pelvic pathology. In this context, MRI serves as a valuable diagnostic tool to help differentiate pelvic vascular congestion from other pathologies such as endometriosis or deep infiltrating fibrosis. Only after these causes are ruled out is imaging for vascular abnormalities, such as PCS, usually pursued. Although pelvic and lower abdominal pain are the most frequent manifestations of PCS, atypical symptoms including neurological involvement such as pudendal or femoral nerve irritation have been reported rarely [4]. The presence of these neurological signs may complicate diagnosis and mimic lumbosacral or gynecologic pathologies.

This case report describes a rare presentation of PCS with image-confirmed irritation of the pudendal and femoral nerves. To our knowledge, few published cases have demonstrated a direct relationship between dilated pelvic veins and neural compression using advanced imaging techniques [4, 5]. The case underscores the value of magnetic neurography and venography in complex pain syndromes and highlights the successful resolution of symptoms following bilateral ovarian vein embolization.

Our goal is to increase awareness of this rare manifestation of PCS among vascular surgeons, neurologists, and radiologists, and to encourage a multidisciplinary diagnostic approach in patients with unexplained pelvic or lower extremity neurologic pain.

## 2. Case Description

A 30-year-old woman presented with persistent, localized pain in the right iliac fossa, radiating anteriorly to the groin and medially to the proximal thigh. The pain had a dull, pressure-like character, was exacerbated by prolonged standing and peaked during menstruation, partially subsiding by the end of the cycle. The patient described a deep pelvic aching sensation and fullness, without paresthesia or motor weakness. She denied left-sided symptoms, dyspareunia, urinary or bowel symptoms, leg heaviness, or visible varicosities. The radiation pattern and pain quality raised suspicion for irritation of the femoral and pudendal nerves.

**Figure 1:**
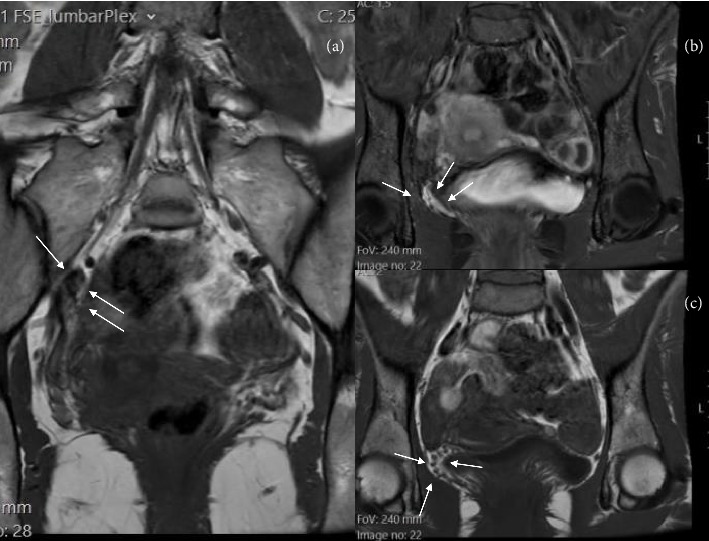
(a) Magnetic neurography shows marked venous engorgement extending into the presacral and paravertebral spaces (white arrows), consistent with pelvic congestion. (b, c) A tortuous vein is seen abutting the right psoas muscle at the level of the L5 nerve root (arrowhead), in close proximity to the origin of the femoral nerve.

Magnetic neurography of the lumbosacral spine revealed prominent pelvic venous engorgement, extending into the paravertebral and presacral spaces. A tortuous vein was observed in direct contact with the inferior aspect of the right psoas muscle, adjacent to the L5 nerve root and close to the origin of the femoral nerve. Although no nerve root compression was present, the findings were consistent with perineural venous irritation and mild displacement of neural structures (Figure 1).

**Figure 2:**
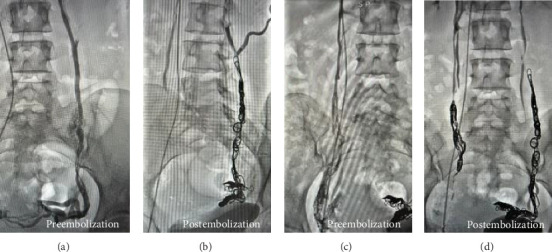
(a) Selective venography of the left ovarian vein demonstrating significant reflux and contrast stasis, consistent with venous insufficiency. (b) Postembolization image of the left ovarian vein showing complete occlusion following coil deployment and sclerotherapy. (c) Venography of the right ovarian vein showing marked insufficiency and reflux into parauterine branches. (d) Postembolization image of the right ovarian vein demonstrating successful occlusion with no residual reflux.

The findings were consistent with PCS, attributed to dilated ovarian veins. Venography confirmed extensive venous insufficiency with significant stasis in parauterine branches, predominantly affecting the right iliac fossa, along with collateral branches from the left iliac crest. Bilateral embolization was performed using Ruby coils (Penumbra, Alameda, CA, USA) and adjunct sclerotherapy. Final venography demonstrated successful occlusion of both ovarian veins without reflux (Figure 2).

### 2.1. Outcome

Postoperative recovery was uneventful with complete resolution of the symptoms. Follow-up imaging and clinical evaluation at 1 year showed no recurrence, and the patient subsequently had a successful pregnancy.

## 3. Discussion

This case highlights an unusual neurological manifestation of PCS in a premenopausal woman. While PCS is primarily recognized as a cause of chronic pelvic pain due to venous insufficiency, neurological symptoms—such as irritation of the pudendal and femoral nerves—are rarely reported [6]. In our patient, advanced imaging with magnetic neurography and venography clearly demonstrated that dilated ovarian and parametrial veins were compressing neural structures, providing essential diagnostic insights. This case highlights the potential for anatomical crowding and venous compression to affect neural structures within the pelvis. The dilated ovarian and collateral pelvic veins likely contributed to perineural engorgement in the presacral space and adjacent to the lumbosacral plexus. Advanced imaging with magnetic neurography provided strong anatomical correlation with the patient's symptoms. The successful embolization of both ovarian veins and associated pelvic collaterals may have resulted in decompression of the perineural venous plexus, explaining the complete symptom resolution.

The anatomical relationship between pelvic veins and lumbosacral nerves is complex and clinically significant. In our case, venous engorgement extended into the presacral and paravertebral regions, where dilated parauterine and paravaginal veins likely exerted pressure on the pudendal and femoral nerves or their proximal branches. These areas are traversed by the perineural venous plexus, which can become congested in PCS. During the procedure, embolization was performed not only on the ovarian veins but also on extensive collateral branches draining into the pelvic and paravertebral venous systems. This comprehensive approach may have led to decompression of the affected neural structures, explaining the rapid and complete symptom resolution.

Although isolated reports have described neurological manifestations such as low back pain, sciatica, or perineal discomfort in PCS, direct compression of specific nerve structures—particularly the pudendal or femoral nerves—has rarely been confirmed with imaging [4, 5]. In contrast to previous literature, our case offers radiological documentation via magnetic neurography and venography, clearly demonstrating venous engorgement compressing neural structures in the lumbosacral area. This sets it apart from most reports where neural involvement was presumed based on clinical symptoms alone. Additionally, few prior reports [4] have demonstrated complete symptom resolution after embolization in cases with documented nerve irritation.

The targeted bilateral embolization using Ruby coils combined with adjunct sclerotherapy resulted in complete symptom resolution and preserved reproductive function, as supported by prior reports on the efficacy of endovascular treatments in PCS [7]. This favorable outcome underscores the potential of minimally invasive therapies in managing complex cases of PCS with neural involvement.

Given the limited literature on neurological complications in PCS, this case emphasizes the importance of considering atypical presentations during diagnosis and treatment. Early recognition and prompt intervention are crucial to prevent chronic nerve injury and optimize overall patient outcomes. Moreover, a multidisciplinary approach involving vascular surgeons, neuroradiologists, and neurologists is essential for comprehensive management [8]. Recent comprehensive reviews have further highlighted advances in the diagnosis and management of PCS, reinforcing the value of precise imaging techniques and targeted therapies in improving patient care [9].

Future research is warranted to elucidate the prevalence and clinical significance of nerve involvement in PCS and to refine imaging and therapeutic techniques for these patients.

## Data Availability

The data that support the findings of this study are available on request from the corresponding author. The data are not publicly available due to privacy or ethical restrictions.
